# Noscapine Inhibiting the Growth and Angiogenesis of Human Eutopic Endometrium of Endometriosis Patients through Expression of Apoptotic Genes and Nitric Oxide Reduction in Three-Dimensional Culture Model

**DOI:** 10.22037/ijpr.2019.1100642

**Published:** 2019

**Authors:** Mohammad Rasool Khazaei, Mohammad Hossein Nasr-Esfahani, Farzaneh Chobsaz, Mozafar Khazaei

**Affiliations:** a *Fertility and Infertility Research Center, Health Technology Institute, Kermanshah University of Medical Sciences, Kermanshah, Iran.*; b *Department of Reproductive Biotechnology, Reproductive Biomedicine Research Center, Royan Institute for Biotechnology, ACECR, Isfahan, Iran.*

**Keywords:** Endometriosis, Noscapine, Apoptosis, Nitric oxide, Sirtuin

## Abstract

Noscapine is a natural alkaloid with anti-angiogenesis activities. The aim of the present study was to examine the effect of noscapine on eutopic endometrium of endometriosis patients (EEE) and normal endometrium (NE) in a three-dimensional (3D) culture model. In this experimental *in-vitro* study, EEE (n = 8) and NE (n = 8) biopsies were taken from 16 reproductive aged women. The biopsies were cleared from blood and mucus. Each biopsy was cut into small fragments (1 × 1 mm) in a sterile condition. For 3D culture, the endometrial fragments were put between two layers of fibrin jell made of fibrinogen solution [3 mg/mL in Medium199 (M199) + thrombin]. Twenty-four wells of culture dish was divided into 5 groups for each biopsy: the control wells were treated with M199 containing 5% fetal bovine serum (FBS) while, the test wells were exposed to the same media containing one of the noscapine doses (10, 50, 100, and 200 μM). The expression of apoptotic genes, growth score, angiogenesis, and nitric oxide (NO) secretion were evaluated. The mean of growth score of groups exposed to 0, 10, 50, 100, and 200 μM were 2.2 ± 0.55, 1.7 ± 0.45, 1.44 ± 0.27, 0.29 ± 0.1, and 0.1 ± 0.08 in EEE, and also, 2.11 ± 0.6, 1.65 ± 0.5, 0.79 ± 0.41, 0.18 ± 0.1, and 0.1 ± 0.1 in NE, respectively, and the difference between the groups was significant (*P* < 0.05). The expression of apoptotic genes significantly increased while, the levels of Bcl-2 and Sirt1 reduced (*P* = 0.004). NO secretion reduced significantly (*P *< 0.05) in both EEE and NE groups. In conclusion, higher doses of noscapine showed inhibitory effect on growth and angiogenesis of EEE and NE.

## Introduction

Human endometrium is unique and special tissue which plays central role in reproduction. During menstrual cycle, endometrium undergoes intensive growth, angiogenesis, and remodeling. Clinically, the presence of endometrial tissue outside endometrial cavity is characterized as endometriosis and its frequency is around 10-15% in fertile and 40% in infertile women (1, 2). 

In fact, endometriosis is a benign cancer-like disorder with abnormal cellular proliferation and angiogenesis due to increased secretion of estrogen hormone. Therefore, it is considered as an angiogenic and estrogen-dependent disease (3). 

In spite of early detection of endometriosis by Sampson at 1927, its pathogenesis and molecular mechanisms remain to be completely elucidated (4). Premier therapy for acute endometriosis is surgery which is usually associated with its recurrence. Therefore, there is a quest for a conservative and irremeably pharmacological approach. In this regards, a number of pharmaceutical agents including statins, letrozole, raloxiphene, and celecoxib have been studied to overcome the pathogenesis and recurrence of endometriosis (5-9). 

Noscapine is a water-soluble alkaloid derived from *Papaver somniferum* (*Papaveraceae *family) which was initially used to treat malaria. It has anti-tussive, anti-cancer, and anti-metastasis properties with low toxicity, as well as the lack of sedative, addictive, and analgesic properties (10). Noscapine showed anti-proliferative effects on prostate, breast, ovarian and cervical cancer cells (11). Aneja* et al. *reported that noscapine could induce apoptosis in colon cancer cells, and our pervious study showed synergic anticancer activity of noscapine with paclitaxel against two human prostate cancer cell lines (12, 13). Furthermore, the survival of noscapine-treated liver cancer cells has been drastically shown to be reduced in a time- and dose-dependent manner with no significant effect on non-cancerous normal cells (14).

Nitric oxide (NO) is an important biological signaling factor in female reproductive processes such as ovulation, menstruation, implantation, pregnancy, and parturition (15). The invovlement of NO synthetase (NOS) as a strong vasodilator and pivotal mediator of angiogenesis has been identified in endometrium and myometrium (16). Steroid hormones as the important adaptors and regulators of NO synthesis play important roles in regulation of endometrial growth and angiogenesis (17). NO also regulates apoptosis so that the low level of NO can lead to apoptosis (18). Apoptosis is also a useful process in conservation of cellular homeostasis in menstrual cycles by omitting aged cells from functional layer of endometrium in menstrual and late secretory phases (19).

Sirtuins are the main regulators of several physiological processes including carcinogenesis, metabolism, and aging. The expression level of sertuins varies in different cancers. They also regulate signaling pathways related to steroid hormones receptors (20). Sirtuin1 is a NAD^+^-dependent acetylase which plays an important role in regulating cellular responses in stress conditions (21). Considering similarity of endometriosis with solid tumors, it seems that the expression level of sirtuin1 changed in EEE. In our previous study, we showed that noscapine inhibits proliferation of isolated stromal and epithelial cells of EEE and NE in primary (two-dimensional) culture (22). Three-dimensional (3D) tissue culture in fibrin matrix is an experimental model for the study of the drug effects on endometriosis which is possible to evaluate the growth and angiogenesis of endometrial explants in a similar *in-vivo* manner. 3D endometrial culture in fibrin matrix was introduced and developed by Casper’s team (5, 23). The purpose of the present study was to investigate the effect of different doses of noscapine on growth, angiogenesis, nitric oxide secretion, and expression of some apoptotic and non-apoptotic genes in EEE and NE using *in-vitro* 3D culture model.

## Experimental

In this experimental in-vitro study, biopsies were obtained from eutopic endometrium of endometriosis (EEE) patients (grade III and IV) (n = 8) during diagnostic laparoscopy. Biopsies of Normal endometrium (NE) were taken from fertile women (n = 8) referring for infertility diagnosis. The women were at reproductive age (25-40 years) and the biopsies were taken at mid-secretory phase of menstrual period, a part of biopsy was used for pathological diagnosis and other part was used in this study. The subjects that received drugs during past 3 months before surgery and also, the subjects with endometrial abnormalities including polyp, cancer, and hyperplasia were excluded from this study. The study was approved by medical ethics committee of Kermanshah University of medical sciences and written informed consent was collected from individuals that participated in this study. 

*Three-dimensional culture*

**Figure 1:**
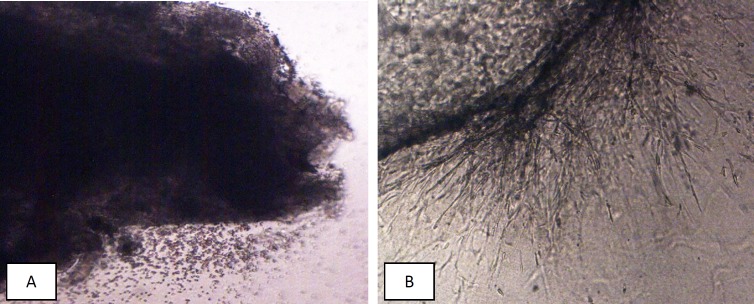
(A) Small endometrial fragment in fibrin jell at first day of 3D culture, there is no morphological changes. (B) Growth changes (endothlial cells sprothig) during 3rd week of 3D culture

3D culture in fibrin matrix was carried out according to the described protocol in our previous studies (5-9). In brief, the biopsies were transferred to laboratory in a cold sterile medium containing antibiotics, and then were washed in new cold PBS containing antibiotic and cleared from residual blood clots and mucus. Each endometrial biopsy was cut into 1 × 1 mm fragments in a sterile petri dish. Tissue culture was performed in one 24-well culture plates for each biopsy. For this aim, 0.5 mL of fibrinogen solution (3 mg/mL in M199) and 15 μL thrombin (Stago, Fardavar Azema, Iran) were added to each well to form first layer of fibrin gel. Then, an endometrial fragment was placed in center of well and covered by an additional 0.5 mL fibrinogen/thrombin solution (Figure 1A).

After formation of the second layer of fibrin gel, each well were supplemented with 1 mL Medium 199 containing antibiotic/antimycotic solution (Sigma) and 5% FBS (Gibco). A drug treatment was performed by addition of noscapine (C22H23NO27, MW = 413.43) at 0 (control), 10, 20, 50, 100, and 200 μM doses (13, 22). The plates were incubated at 37 ºC in 5% CO_2_ in a humidified environment. The media were replaced each three days and the supernatants were separately isolated and preserved at -20 °C. 

In days of 7, 10, 15, and 21, the proliferation of epithelial and stromal cells, the angiogenesis and formation of monolayer epithelial cell were evaluated by two independent researchers via scoring method, and mean of these data were recorded (7, 9). The growth of each fragment was graded from 0 to 4 in which zero is no growth development in tissue explants, 1 is the presence of minute sprouts of endothelial and epithelial cells in less than 25% of each fragment, 2 is the same growth development in 26-50% of each fragment, 3 is the growth development in 51-75% and 4 is the growth development (Figure 1B) in more than 75% of tissue fragments. The growth scores of days 7, 10, 15, and 21 were used for data analysis.

**Figure 2:**
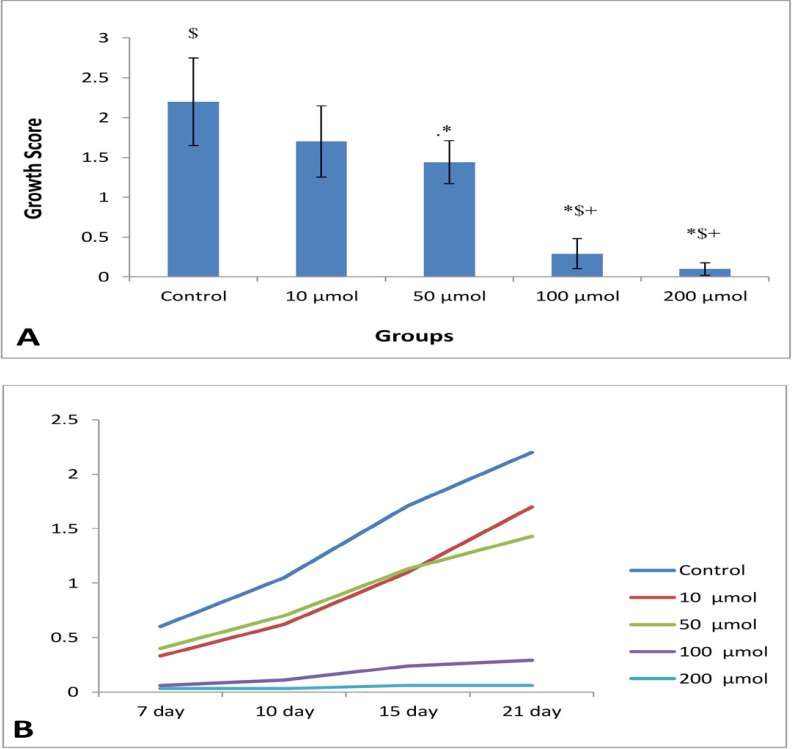
(A) Mean of growth score of EEE (n = 8) in control and different doses of noscapine at the end of study periods (21th days). Significant difference: (*) whit: control, (+) whit 10 μM, and ($) whit 50 μM. (B) Process of endometrial growth during 21 days in control and different doses of noscapine

**Figure 3:**
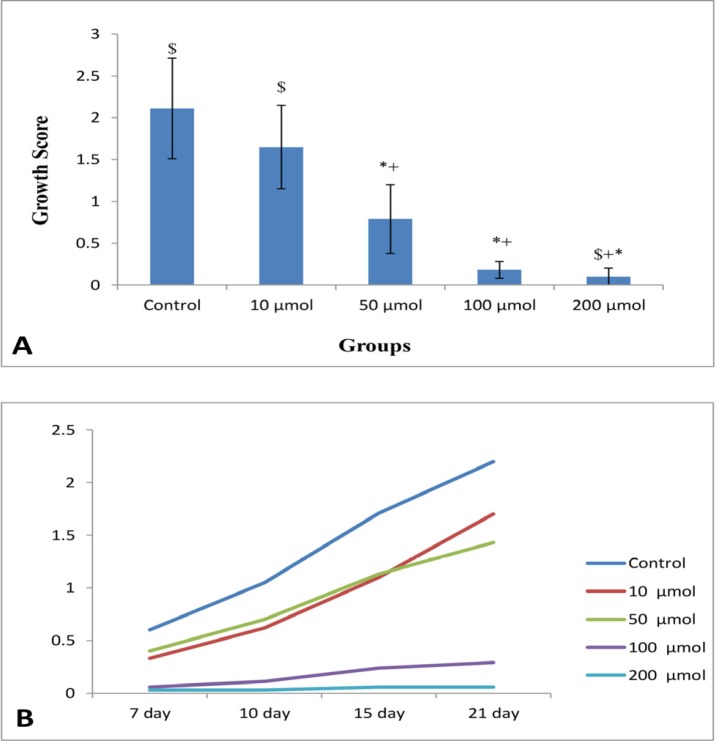
(A) Mean growth score of NE (n = 8) in control and different doses of noscapine. Significant difference: (*) whit control, (+) whit 10 μM, and ($) whit 50 μM. (B) Process of endometrial growth during 21 days 3D culture in control and different doses of noscapine

**Figure 4:**
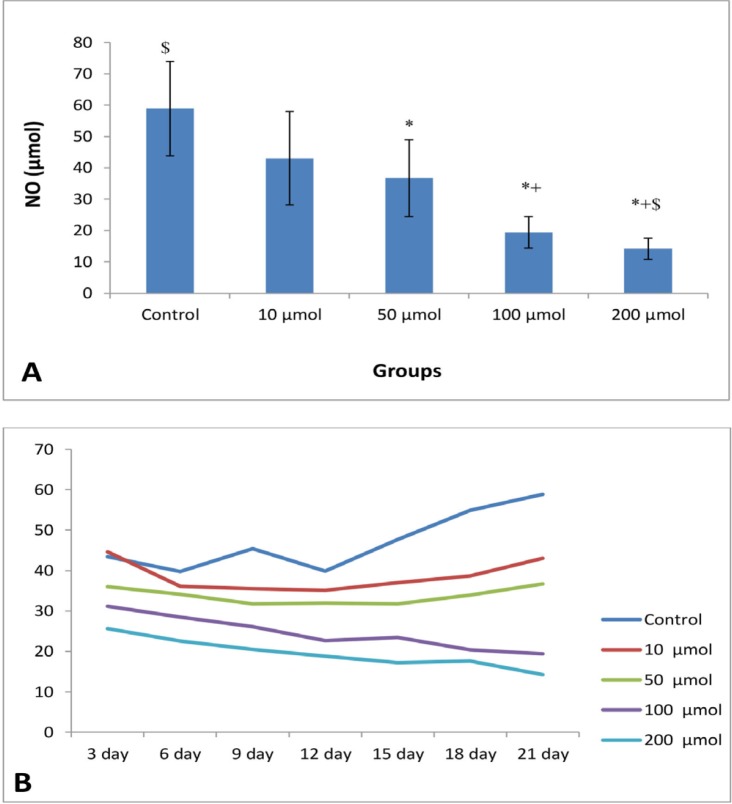
(A) Mean of NO levels (μM) of EEE (n = 8) in control and different doses of noscapine. Significant difference: (*) with control, (+) whit 10 μM, and ($) whit 50 μM. (B) Process of NO levels in control and different doses of noscapine during 21 days 3D culture

**Figure 5:**
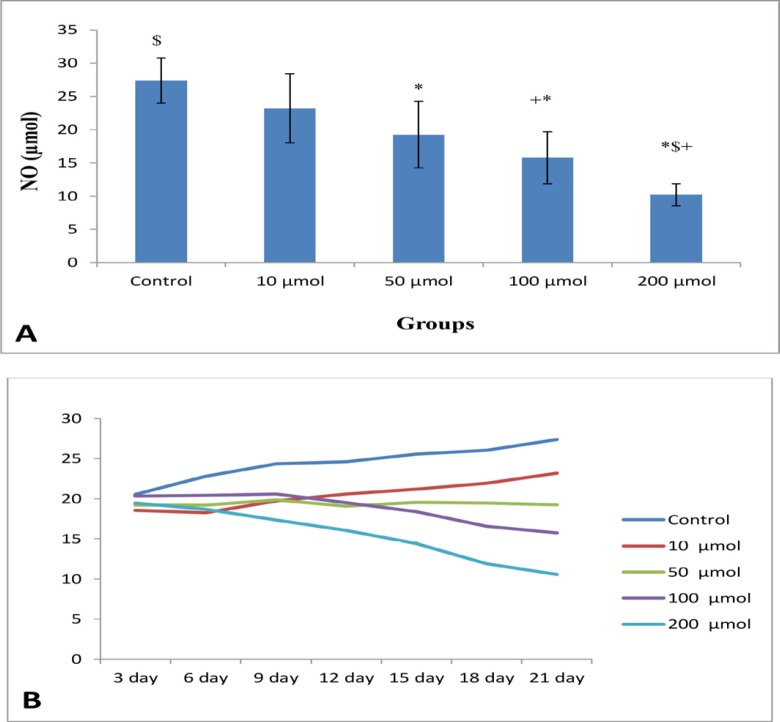
(A) Mean of NO levels (μM) of NE (n = 8) in control and different doses of noscapine. Significant difference: (*) whit control, (+) with 10 μM, and ($) with 50 μM. (B) Process of NO levels (μM) in control and different doses of noscapine during 21 days 3D culture

**Figure 6:**
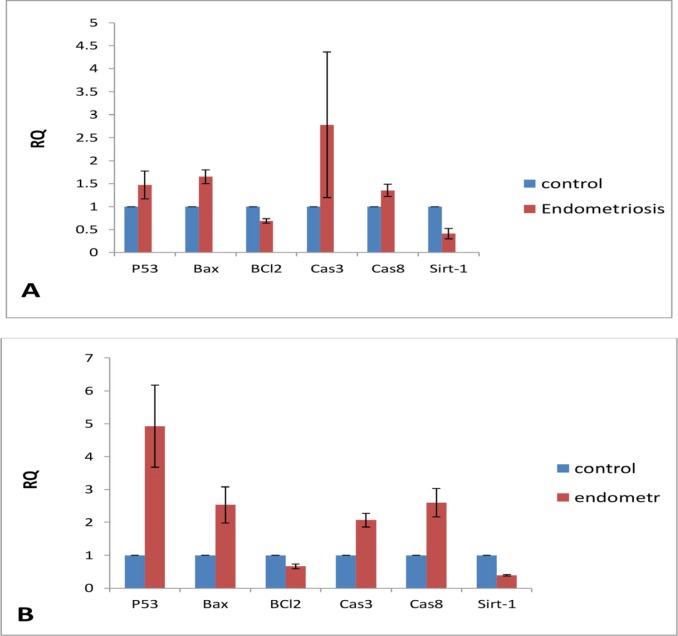
(A) The expression of p53, Bax, BCl2, caspase 3, caspase 8 and Sirt-1 in EEE cells and (B) NE cells exposed to 200 μM noscapine

*Nitric Oxide assay*

Nitric oxide (NO) was measured by Griess method (23). In view of the instability and low half-life of NO, the concentration of nitrate as an oxidative metabolite of NO is measured in collected supernatant of each well during replacement of media at each three days of incubation. In this method, the increased doses of sodium nitrate including 3.25, 6.25, 12.5, 25, 50, 100, and 200 μM were used as standard solutions. For deproteinization of supernatant, zinc sulfate was added to each supernatant and centrifuged. Next, vanadium chloride was mixed with supernatant to convert nitrite into nitrate. Then, Griess reagent, mixture of sulfonamide 0.2% and naphthylendiamine dihyrochloride (NEED) 0.1% was added to all standard and experiments microplates and incubated at 37 ºC to appear a serial purple colure in standard wells (20-30 min). The absorbance of each well was determined at 540 and 650 nm using an ELISA reader (Statfax 100, USA).

*RNA extraction*

EEE and NE cells were isolated (8, 23) and cultured at 37 ºC. When the number of cells reached to the appropriate level, medium containing 200 μM noscapine was added. After 72 h, the cells were collected by 1 mL RNX-PLUS, transferred to RNase-free tubes, mixed with 200 μM cold cholorophorm for 5 min and centrifuged at 12000 rpm for 20 min. The supernatants were transferred to a new tubes and was filled with an equal volume of cold ethanol (100%), They were shaking and placed in 20 ºC for 20 min. Then, the tubes were centrifuged at 12000 rpm for 15 min. The supernatant was discarded and 1 mL cold ethanol (75%) was added to pellet and centrifuged at 7500 rpm for 8 min. The supernatant was slowly discarded to prevent displacement of pellet (24). The pellets were incubated to dry and 30-50 μL of DNA-RNA free distilled water was added to dissolve pellets. The amount of RNA was measured using a nanodrop. 

*Real-Time PCR*

About 500 ng of each extracted RNA were reverse transcribed into cDNA with the cDNA synthesis kit (PrimeScriptTM cDNA Synthesis Kit, Takara), according to the manufacturer’s instructions. The resulting cDNA was kept at −20 °C until use. The expression profile of P53, BAX, BCL-2, CASPASE 3, CASPASE 8, SIRT 1, and β-ACTIN (as an internal control) genes, were evaluated using Real-Time PCR based SYBR GREEN I assay (SYBR Premix Ex Taq Master Mix, Takara). Real time PCR was carried out using Applied Biosystems™ Real-Time PCR instruments. Ten µL of SYBR Green Master Mix, 2 µL of cDNA, and 200 nM of each primer set were used for amplification in 20 µL reaction mixtures. All samples were amplified in triplicates; the cycling conditions were as follows: 10 sec at 95 °C, and 40 cycles at 95 °C for 5 sec and 60 °C for 30 sec. The primer sequences genes are as follow:

P53 (F: 5’-taacagttcctgcatgggcggc-3’, R: 5’-aggacaggcacaaacacgcacc-3’), 

BAX (F: 5’-cctgtgcaccaaggtgccggaact-3’, R: 5’-ccaccctggtcttggatccagccc-3’), 

Bcl2 (F: 5’-ttgtggccttctttgagttcggtg-3’, R: 5’ ggtgccggttcaggtactcagtca-3’), 

Sirt 1 (F: 5’-tcagtgcatggttcctttgc -3’, R: 5’-gttcatcagctgggcaccta -3’), 

Caspase 3 (F: 5’-caaactttttcagaggggatcg -3’, R: 5’-gcatactgtttcagcatggcac -3’),

 Caspase 8 (F: 5’-ggatggccactgtgaataactg -3’, R: 5’-tcgaggaccatcgctctctca -3’) and 

 Β-Actin (F: 5’ agagctacgagctgcctgac -3’, R: 5’-agcactgtgttggcgtacag -3’).

*Statistical analysis*

Data were evaluated by one-way ANOVA and nonparametric tests (Tukey at 5% significance level) using SPSS software (version 16) to compare the difference between groups. The results were expressed as mean ± SEM and *P *< 0.05 was considered significant.

## Results

*Growth score*

At the end of the study period (21^th^ day) (Figure 1B), the mean of growth score of EEE were 2.2 ± 0.55, 1.7 ± 0.45, 1.44 ± 0.27, 0.29 ± 0.1, and 0.1 ± 0.08 in control (0), 10, 50, 100, and 200 μM noscapine respectively, and a significant difference (*P *< 0.05) was observed between the treated groups (except 10 μM dose) compared to the control group. The growth inhibition of EEE was dose-dependent and endometrial growth was almost completely blocked at 100 and 200 μM doses of noscapine (Figure 2A). Furthermore, to determine growth process, as depicted in Figure 1B, the results clearly manifested that the growth of EEE in control, 10 and 50 μM increased in a time-dependent manner with a significant difference (*P *< 0.001) from day 7 to day 21, while such a trend was not observed at 100 and 200 μM doses of noscapine.

The mean growth scores of NE at the end of tissue culture (21^th^ days) in control (0), 10, 50, 100, and 200 μM noscapine were 2.11 ± 0.6, 1.65 ± 0.5, 0.79 ± 0.41, 0.18 ± 0.1, and 0.1 ± 0.1 respectively, with a significant difference between the groups (*P* < 0.01). Data clearly showed that growth inhibitory effect of noscapine was dose-dependent (Figure 3A). In statistical evaluation, the control group showed no difference with the group exposed to 10 μM noscapine while both of them had significant difference with other groups (*P *< 0.001). Evaluation of endometrial growth during 3 weeks of 3D culture indicated that there is a time-dependent increase in growth of tissues in control and 10 μM (*P *< 0.001). However, the groups treated with 100 and 200 μM doses had a steady growth inhibition during 3 weeks (Figure 3B).

*NO levels*

The mean of NO levels of EEE treated with 0, 10, 50, 100, and 200 μM were 58.88 ± 15.06, 43.02 ± 14.86, 36.09 ± 12.27, 19.43 ± 4.99, and 14.20 ± 3.34, respectively with significant difference (*P *< 0.003) (Figure 4A). There are significant differences between control group with doses of 50, 100, and 200 μM, and 10 μM doses with 100 and 200 μM doses (*P* < 0.001). The decreased in NO production in cells treated with 100 and 200 μM doses were relatively constant (Figure 4B). 

The mean of NO levels in NE exposed to 0, 10, 50, 100, and 200 μM were 27.4 ± 3.42 23.19 ± 5.27, 19.24 ± 5.02, 15.77 ± 3.89, and 10.57 ± 1.65, respectively (significant difference, *P *< 0.01) (Figure 5A). The level of NO indicated a time-dependent reduction in test groups compared to the control group. The results clearly revealed that the control and 10 μM groups possessed a relatively time-dependent enhancement, while 100 and 200 μM showed a time-dependent reduction of the level of NO (Figure 5B).

*Gene expression*

After several repeats with different doses of noscapine, the expression of genes at the dose of 200 μM was considered in final assay of gene expression (Figure 6A). In NE, the expression of P53, BAX, CASPASE 3, and CASPASE 8 were increased in 1.5, 1.6, 2.7, and 1.3 folds, respectively, compared to the control group. In addition, the expression of BCL-2 and SIRT 1 was decreased (Figure 6B). The expression of P53, BAX, CASPASE 3, and CASPASE 8 in NE being exposed to 200 μM, respectively, showed the increase in 5, 2.5, 2, and 2.5 folds compared to untreated control, while the levels of BCL-2 and SIRT1 were decreased.

## Discussion

To our knowledge this is the first report of noscapine effect on human endometrial tissue. Noscapine, an anti-tussive, anticancer, and nontoxic natural compound showed promising effect on endometrium of endometriosis patients (EEE) cultured in a 3D model of endometriosis. Therapeutic strategies of endometriosis treatment are mainly based on ovarian suppression by oral contraceptives, androgenic agents and progestin, or surgery, but recurrence of disease associated with surgical intervention, and unwanted side-effects often are associated with hormonal treatments. So, seeking new drugs are needed for regression of disease and its symptoms without adverse hypo-estrogenic effects (25).

Higher doses of Noscapine (100 and 200 μM) exhibited a complete suppression of endometrial growth and angiogenesis in both EEE and NE. In our previous 2-dimensional study, the growth inhibitory effects of noscapine on isolated stromal and epithelial cells of EEE and NE were documented in a dose-dependent manner and the same effects were confirmed in 3D culture (23). As we cannot use human tissue in many studies, the researchers believed that 3D culture models have better condition than 2D culture and fibrin matrix in our study mimic the *in-vivo* condition in endometriosis disease (25).

One of the new findings of the present study is reduction of SIRT1 expression in EEE and NE by noscapine. SIRT1 is one of the sirtuin families which is the main regulators of carcinogenesis. The expression of sertuins varies in different cancers. They also regulate signaling pathway of receptors of steroid hormones (20). Sirtuin1 plays a pivotal role in regulating cellular responses in stress conditions (21). Considering similarity of endometriosis with solid tumors, it seems that the expression of SIRT1 changes in EEE compared to NE. SIRT1 is a main stimulator of cell growth and angiogenesis, two main events in endometriosis. The evidences showed that SITR1 modulating could be beneficial in several diseases such as endometriosis and cancer. In the present study, noscapine suppressed SIRT1 expression, cell proliferation and angiogenesis, and NO secration of endometriotic explants.

Noscapine exerts different effects on the biopsies of EEE and NE, it increased expression of P53, BAX, CASPASE 3, and CASPASE 8, while decreased the levels of BCL-2 and SIRT1. Also, Caspase 3 expression was more prominent in EEE while, P53 expression was higher than other genes in NE. The endometrium from normal and endometriosis patients has differences in molecular biology, and some characteristics of EEE are resembled to cancerous tissue. The apoptotic effects of high doses of noscapine were investigated in cancer cell lines, including human and animals, but anti-cancer effect of noscapine was not determined completely; however, it seems that this activity is mediated by several mechanisms, such as induction of apoptosis (14, 26), expression of P53 and P21, as well as suppression of BCL-2 and antitubuline properties (12, 27). 

Noscapine effect on EEE and NE was dose dependant, although comparison of these tissues indicated that controls of EEE showed a better growth score than NE (2.2 *vs.* 2.1). High doses of noscapine (200 and 100 μM) exert same anti-proliferating effect on both endometrial samples (EEE and NE) during the study course. Although, low doses of noscapine (10 μM) did not show any significant inhibitory effect compared to the control group, there is a considerable difference at 50 μM dose of noscapine, which showed a stronger inhibitory effect on EEE than NE (0.79 *vs.* 1.5).

In biology and pathology of reproductive system organs such as uterus, NO is an important regulator. A number of studies have demonstrated the increased variation in the expression of NOS; however, the regulating factors of NO production in the uterus remain to be clear (28). Previous studies reported that NO changed in endometriosis, and in the present study, NO secretion by EEE were higher than NE implying the increased production of NO in the endometriosis patients (23, 29). In our study, the inhibitory effect of higher doses of noscapine is mediated by reduction of NO level.

Noscapine is an old anti-cough alkaloid from opium with low toxicity and suitable tolerance discovered by Joshi’s team. It is a potent anti-tumor agent with very few side effects drawing considerable attention (30). Noscapine induced mitotic arrest and apoptosis by binding to microtubule in the mammalian cells. In conclusion, according to similarity between solid tumor and endometriosis in cell proliferation, apoptotic pathway, some gene expression and angiogenesis, noscapine, a water soluble natural compound without sedative, addictive and analgesic properties could be suggested as a new drug for treatment of endometriosis. 
